# Anogenital pruritus is associated with anxiety, depression, and other psychiatric disorders

**DOI:** 10.1097/JW9.0000000000000168

**Published:** 2024-07-10

**Authors:** Alex Balfour, Christina Kraus

**Affiliations:** a University of California Irvine, School of Medicine, Irvine, California; b Department of Dermatology, University of California, Irvine, California

**Keywords:** anogenital pruritus, anxiety, depression, psoriasis, vulvar pruritus

**What is known about this subject in regard to women and their families?**

- Anogenital pruritus is a condition that overwhelmingly affects women and can be debilitating to quality of life.

**What is new from this article as messages for women and their families?**

- There is evidence that anogenital pruritus is associated with mood disorders.
- Awareness of this association is important for clinicians to provide optimal and comprehensive care for patients suffering with this condition, and their families.

Chronic pruritus has been shown to be associated with decreased quality of life, psychological stress, depression, and anxiety.^1^ While anogenital pruritus (AP) is common, it is an often undisclosed symptom of various dermatologic, neurologic, and systemic conditions. Multiple studies have evaluated the effects of other forms of localized or generalized pruritus on quality of life; however, there are fewer studies examining AP. AP has been associated with stigmatization experiences and sexual dysfunction.^2^ One study found that AP is one of the most common and debilitating symptoms of genital psoriasis.^3^ Herein, we aimed to evaluate the association between AP and psychiatric disorders by performing a population-level retrospective case–control study.

We utilized TriNetX, a global health research network of over 100 million patients, to search International Classification of Diseases, 10th revision (ICD-10) codes L29.0 to L29.3 indexed within the last 20 years. AP was considered the index event. Psychiatric disorders included in the analysis are depressive episode, major depressive disorder, persistent mood disorder, bipolar disorder, unspecified mood disorder, anxiety disorder, and reaction to severe stress and adjustment disorders (ICD-10, F30–F48). A majority of patients had a diagnosis of major depressive disorder (F33), depressive episode (F32), or other anxiety disorders (F41). Control cohorts were 1:1 propensity matched by age, sex, and race/ethnicity and patients with a diagnosis of AP were excluded. Control cohorts consisted of the final diagnoses: encounter for general adult examination (Z00.0) or psoriasis (L40). Patients undergoing general examinations were chosen to represent a general population of patients without active concerns. While there is overlap between AP and other dermatologic conditions, we chose to also compare with a cohort of psoriasis patients, as psoriasis has been known to be associated with increased rates of depression and anxiety.^4,5^

We identified 286,109 patients with AP and an equal number of matched controls (Table 1). Patients with AP have increased odds of psychiatric disorders when compared to patients with a general adult examination (1.323, 1.309–1.336) or psoriasis (1.234, 1.221–1.248). These findings suggest that AP is associated with psychiatric disorders, even when compared to matched cohorts of psoriasis, which has been independently associated with increased rates of depression and anxiety.^4^ Reaction to severe stress and adjustment disorders, persistent mood disorders, and unspecified mood disorders had the highest odds ratio within both control groups. For general adult medical examinations, bipolar disorder had the second highest, while for psoriasis this was anxiety disorder. There was a significant difference in the association between either gender with AP and the psychiatric conditions measured (Table 2).

**Table 1:** Control cohorts “encounter for general medical examination” and “psoriasis” were propensity matched to the cohort of patients with anogenital pruritus

Characteristic	No. (%)	No. (%)	No. (%)	No. (%)
	Anogenital pruritus (*n* = 286,109)	Encounter for general medical examination (*n* = 286,109)	Anogenital pruritus (*n* = 245,051)	Psoriasis (*n* = 245,051)
Age, mean (SD)	41.7 (20.4)	41.7 (20.4)	49.8 (20.4)	49.8 (20.4)
Female	227,297 (79.444)	227,297 (79.444)	188,881 (77.078)	188,881 (77.078)
Male	57,713 (20.172)	57,713 (20.172)	55,240 (22.542)	55,240 (22.542)
Unknown gender	1098 (0.369)	1098 (0.369)	930 (0.38)	930 (0.38)
Not Hispanic or Latino	186,859 (65.31)	186,859 (65.31)	160,910 (65.664)	160,910 (65.664)
Unknown ethnicity	62,989 (22.016)	62,989 (22.016)	56,905 (23.222)	56,905 (23.222)
Hispanic or Latino	36,261 (12.674)	36,261 (12.674)	27,236 (11.114)	27,236 (11.114)
White	173,491 (60.638)	173,491 (60.638)	161,525 (65.915)	161,525 (65.915)
Other race	50,760 (17.741)	50,760 (17.741)	45,289 (18.481)	45,289 (18.481)
Black or African American	48,115 (16.817)	48,115 (16.817)	26,655 (10.877)	26,655 (10.877)
Asian	11,743 (3.948)	11,743 (3.948)	10,142 (4.139)	10,142 (4.139)
American Indian or Alaska Native	1151 (0.387)	1151 (0.387)	803 (0.328)	803 (0.328)
Native Hawaiian or Other Pacific Islander	843 (0.283)	843 (0.283)	637 (0.26)	637 (0.26)

Each characteristic of the control cohort is equivalent to the characteristics of the cohort of patients with anogenital pruritus that was compared to that particular control.

SD, standard deviation.

**Table 2:** Odds ratio, 95% confidence interval, and *P* values for the diagnosis of psychiatric conditions in anogenital pruritus group compared to control groups: encounter for general medical examination (Z00.0) and psoriasis (L40)

Psychiatric disorder	All sexes			Females only			Male only		
	Odds ratio	Confidence interval	*P* value	Odds ratio	Confidence interval	*P* value	Odds ratio	Confidence interval	*P* value
Odds ratio for diagnosis of psychiatric conditions in AP group compared to general medical examination control cohort									
Depression	1.4	1.383–1.418	<.0001	1.43	1.410–1.450	<.0001	1.274	1.230–1.319	<.0001
Anxiety	1.285	1.271–1.300	<.0001	1.284	1.268–1.301	<.0001	1.31	1.270–1.351	<.0001
Bipolar	1.571	1.521–1.623	<.0001	1.644	1.584–1.705	<.0001	1.357	1.239–1.487	<.0001
Other*	1.651	1.626–1.678	<.0001	1.744	1.714–1.776	<.0001	1.422	1.361–1.486	<.0001
All	1.323	1.309–1.336	<.0001	1.344	1.328–1.360	<.0001	1.274	1.241–1.308	<.0001
Odds ratio for diagnosis of psychiatric conditions in AP group compared to psoriasis control cohort									
Depression	1.186	1.170–1.201	<.0001	1.24	1.222–1.258	<.0001	1.044	1.009–1.080	<.0001
Anxiety	1.242	1.227–1.257	<.0001	1.275	1.258–1.293	<.0001	1.264	1.225–1.304	<.0001
Bipolar	1.083	1.048–1.119	<.0001	1.151	1.110–1.194	<.0001	0.909	0.837–0.989	<.0001
Other^a^	1.459	1.435–1.484	<.0001	1.564	1.534–1.593	<.0001	1.297	1.241–1.356	<.0001
All	1.234	1.221–1.248	<.0001	1.286	1.270–1.303	<.0001	1.169	1.138–1.201	<.0001

AP, anogenital pruritus.

a Other disorders refer to “reaction to severe stress and adjustment disorders (F43),” “persistent mood (affective) disorders (F34),” and “unspecified mood (affective) disorders (F39).”

Overall, we found a significant association between AP and psychiatric disorders. Limitations to our study include the retrospective nature and use of ICD-10 codes that may lead to misdiagnosis. Additionally, we could not evaluate the etiology of AP that may independently contribute to psychiatric disorders. These findings highlight the importance of evaluating patients with dermatologic conditions for AP and considering screening for psychiatric disorders as appropriate. Future studies are necessary to better understand the incidence of AP in other skin conditions and evaluate additional factors that may contribute to psychiatric disorders in patients with AP.

## Conflicts of interest

None.

## Funding

None.

## Study approval

N/A

## Author contributions

All authors participated in research design, data analysis, and writing of the paper.
